# UGT74B5-mediated glucosylation at *ortho* hydroxyl groups of benzoic acid derivatives regulating plant immunity to anthracnose in tea plants

**DOI:** 10.1093/hr/uhaf009

**Published:** 2025-01-14

**Authors:** Caiyun Li, Feixue Wu, Lei Yang, Nana Liu, Xinfu Zhang, Fengfeng Qu, Liping Gao, Tao Xia, Lei Zhao, Peiqiang Wang

**Affiliations:** College of Horticulture, Qingdao Agricultural University, No. 700 Changcheng Road, Chengyang District, Qingdao, Shandong, 266109, China; College of Horticulture, Qingdao Agricultural University, No. 700 Changcheng Road, Chengyang District, Qingdao, Shandong, 266109, China; College of Horticulture, Qingdao Agricultural University, No. 700 Changcheng Road, Chengyang District, Qingdao, Shandong, 266109, China; College of Horticulture, Qingdao Agricultural University, No. 700 Changcheng Road, Chengyang District, Qingdao, Shandong, 266109, China; College of Horticulture, Qingdao Agricultural University, No. 700 Changcheng Road, Chengyang District, Qingdao, Shandong, 266109, China; College of Horticulture, Qingdao Agricultural University, No. 700 Changcheng Road, Chengyang District, Qingdao, Shandong, 266109, China; School of Life Science, Anhui Agricultural University, No. 130, Changjiang West Road, Shushan District, Hefei, Anhui 230036, China; State Key Laboratory of Tea Plant Biology and Utilization, Anhui Agricultural University, No. 130 Changjiang West Road, Shushan District, Hefei, Anhui 230036, China; College of Horticulture, Qingdao Agricultural University, No. 700 Changcheng Road, Chengyang District, Qingdao, Shandong, 266109, China; College of Horticulture, Qingdao Agricultural University, No. 700 Changcheng Road, Chengyang District, Qingdao, Shandong, 266109, China

## Abstract

Benzoates, particularly salicylic acid (SA) and its derivatives, play critical roles in plant immune responses and basal defense through hydroxylation and glycosylation. Anthracnose is one of the most common and devastating diseases in tea plants (*Camellia sinensis*). However, the role of SA and its derivatives in tea plant immunity and resistance to anthracnose remains largely unexplored. In the present study, we identified and characterized a glycosyltransferase, CsUGT74B5, which was significantly downregulated in tea seedlings upon anthracnose infection. *CsUGT74B5* was preferentially expressed in mature leaves and stem, and responded rapidly to exogenous SA treatment. Phylogenetic analysis suggested CsUGT74B5 might possess the catalytic activity toward benzoates. Enzymatic assays and molecular docking demonstrated recombinant CsUGT74B5 specifically glycosylated at the *ortho* hydroxyl groups of SA and 2, 6-dihydroxybenzoic acid (2, 6-DHBA), but did not glycosylate 2, 3-DHBA, 2, 5-DHBA, or other substrates *in vitro*. Overexpression of *CsUGT74B5* in *Arabidopsis thaliana* and tobacco (*Nicotiana tabacum*) reduced SA level while promoting the accumulation of SA 2-*O*-β-D-glucoside (SAG), further validating the *in vivo* function of CsUGT74B5. Moreover, transient overexpression of *CsUGT74B5* in two tea plant cultivars increased their sensitivity to anthracnose and accelerated lesion development, which was attributed to decreased SA levels. Overall, our finding demonstrated that CsUGT74B5-mediated biosynthesis of SAG regulated tea plant immunity against anthracnose by fine-tuning free SA levels, providing new progress into the immunity response of tea plants.

## Introduction

**Figure 1:**
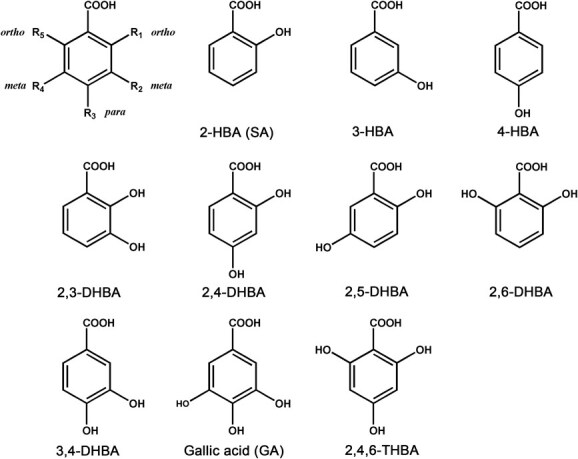
Chemical structural formulas of benzoates.

Benzoates are one class of natural secondary metabolites with significant industrial and biological importance, typically found in either free form or as conjugates with other molecules, such as glucose, attached to hydroxyl or carboxyl groups on the benzene ring [1]. Naturally occurring benzoate derivatives carry hydroxyls at various positions relative to the carboxyl, including the *ortho* position (2-OH, 6-OH), *meta* position (3-OH, 5-OH), and *para* position (4-OH) (Fig. 1). The position and numbers of hydroxyl groups on the benzene ring are key determinants of the compound’s properties. Benzoic acid and its derivatives often exhibit antibacterial and antiinflammatory effects, which are crucial for protecting plants from invasion and infection by exogenous pathogens. For instance, the surface of tea leaves can secrete protocatechuic acid (3, 4-DHBA) and some other phenolic compounds that have inhibitory effects on various pathogens, among which 3, 4-DHBA specifically inhibits the germination of tea anthracnose pathogen *Gloeosporium theae*-*siliensis* [2]. Additionally, para-hydroxybenzoic acid (4-HBA) acts as an allelochemicals in soil, potentially contributing to crop succession obstacles [3].

SA, also known as 2-hydroxybenzoic acid (2-HBA), is one of the most common benzoate derivatives in plants. As a multifaceted phytohormone, SA regulates plant responses to both abiotic and biotic stresses [4]. SA and its derivative are known to activate disease resistance mechanisms in plants, including pathogen-associated molecular pattern-triggered immunity (PTI) and effector-triggered immunity (ETI) in response to pathogen invasion ( [5–8]. Systemic acquired resistance (SAR), a widespread inducible immune mechanism in plants, requires the accumulation of SA for its expression [9, 10]. Depletion of SA would abolish local pathogen resistance, systemic and local induction of PR genes, and SAR development [9, 11]. In plants, SA can be further modified and transformed into various forms for utilization, including hydroxylation, glycosylation, methoxylation, and so on [12, 13] [14, 15]. DHBAs such as 2, 3-DHBA and 2, 5-DHBA, which carry two hydroxyls on the benzene ring, are synthetized through hydroxylation of SA. These derivatives can improve plant defense, induce PR genes, and participate in leaf senescence through modulating the SA homeostasis [14, 16, 17]. In tomato and rice, disruption of the *salicylic acid 5-hydroxylase* (*S5H*) ortholog promoted SA accumulation and enhanced broad-spectrum pathogen resistance [14, 16].

Glycosylation is another important and common metabolic process in plants, known to regulate the properties of the aglycones, including their bioactivity, solubility, and transport properties. Numerous secondary metabolites (flavonoids and volatiles), plant hormones (IAA, IBA and ABA), and some xenobiotics can be glycosylated under different conditions. UDP-glycosyltransferases (UGTs) are key enzymes in this process, catalyzing the transfer of a glycosyl moiety from an activated donor to the aglycones. To date, several plant SA or its derivative glycosyltransferases have been identified. The glucosylated forms of SA include SA 2-*O*-β-D-glucoside (SAG) and glucosyl salicylate (GS), an ester form. SAG is the predominant stable metabolite of SA in plants, while GS is a high-energy but relatively minor metabolite [11, 18]. In healthy plants, GS is almost undetectable in healthy plants, and it always served as the biosynthetic intermediate of other metabolites. The first SA UGT gene was cloned and validated in tobacco, where the recombinant enzyme catalyzed the generation of GS and SAG in nearly equal proportions [12]. In *Arabidopsis thaliana*, AtUGT74F1 catalyzes the formation of SAG, while AtUGT74F2 preferentially converts SA into GS [19, 20]. Plant SA-associated immune responses also can be modulated through the glycosylation of dihydroxybenzoic acids (DHBAs). In *A. thaliana*, AtUGT76D1 was reported to glycosylate 2, 3(5)-DHBAs to their glucose and xylose conjugates, but failed to glycosylate SA; overexpression of *AtUGT76D1* increased SA biosynthesis and enhanced the immune response pre- and postpathogen infection [21]. Similarly, CsUGT95B17 catalyzed the biosynthesis of 2, 4-DHBA glucoside, which positively regulated the resistance of tea plants when facing the invasion of exogenous pathogens [22].

Tea plants are rich in various secondary metabolites, including polyphenols, caffeine, and theanine, which endowed tea with unique flavor and health benefits [23]. However, tea plant diseases, particularly fungal infections, significantly affect tea quality and yield, leading to substantial economic losses. Among these, anthracnose, caused by *Colletotrichum camelliae* fungal, is one kind of prevalent disease particularly in warm and humid areas. Recently, CsGSTU45, a glutathione S-transferase was found to increase the susceptibility of tea plant to anthracnose by accumulating large amounts of H_2_O_2_ [24]. A ‘pink ring’ often appeared outside the lesions when the tea leaves were inoculated with anthracnose, which was identified to be anthocyanin-3-*O*-galactosides, one kind of anthracnose-resistant phytoalexin [25].

Benzoates play vital roles in plant immune responses through hydroxylation and glycosylation. However, the role of benzoic acid and its derivatives, such as SA and DHBAs, in resisting tea anthracnose has been scarcely studied. In the present study, we identified and characterized a glycosyltransferase, CsUGT74B5, that was downregulated following anthracnose infection. The recombinant CsUGT74B5 displayed specific glucosylation at *ortho* hydroxyl groups of benzoic acid, but failed to glycosylate the *meta* and *para* hydroxyl groups. Overexpression of *CsUGT74B5* in plants increased their sensitivity to exogenous pathogens by lowering endogenous SA levels. Overall, our findings demonstrated CsUGT74B5-mediated biosynthesis of glycosides of SA and 2, 6-DHBA, fine-tuned SA levels, and coordinated immune response in tea plants.

## Results

### Identification of UGTs in response to anthracnose in tea plants

**Figure 2:**
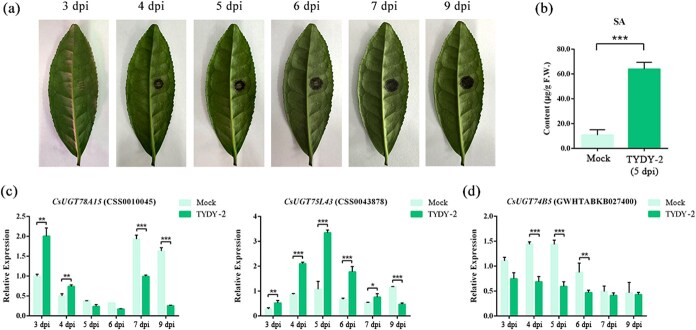
The accumulation profile of SA and expression levels of candidate *UGT* genes during the infection of *C. camelliae.* (a) The phenotype of tea leaves after being inoculated with anthracnose pathogen (TYDY-2). (b) The contents of SA in the leaves of tea plants infected and uninfected with TYDY-2. (c) The expression levels of reported UGT genes *CsUGT78A15* and *CsUGT75L43* in the leaves during the infection of anthracnose. (d) The relative expression levels of the candidate UGT *CsUGT74B5* during the infection of anthracnose. All data presented here are the means of three replicates. Significant differences analysis of data was calculated by one-way analysis of variance (ANOVA) using SPSS 21.0. ^*^P < 0.05, ^**^P < 0.01, ^***^P < 0.001. TYDY-2 is the name of *C. camelliae* strain.

To explore the response of tea plants to anthracnose, we inoculated tea leaves with the pathogenic isolated *C. camelliae* strain (TYDY-2) (Fig. 2a). SA, as an important signaling molecule for plants to respond to biotic and abiotic stresses, can induce plant resistance to these stresses. Five days postinoculation (dpi), the content of SA significantly increased in the leaves surrounding the infected lesions (Fig. 2b). Glycosylation, a crucial modification in plant secondary metabolism, enables plants to cope with various environmental stresses. Numerous genes in tea plants, including several UGTs, were differentially expressed in response to anthracnose. Compared with mock-inoculated leaves, the expression level of *CsUGT78A15* in TYDY-2 inoculated leaves was significantly upregulated on the third and the fourth day, downregulated in later stage. *CsUGT75L43* was significantly upregulated during the period from the third to the seventh day, downregulated in later stage. *CsUGT78A15* and *CsUGT75L43* were previously reported to be involved in the formation of the ‘pink ring’ (the main components of ‘pink ring’ were anthocyanin galactosides) [25] (Fig. 2c). Previously reported UGT members located in Group L catalyzed the glycosidation of benzoates and were involved in plant disease resistance [1, 26]. In this study, a UGT gene named *CsUGT74B5* was significantly downregulated during the infection process of *C. camelliae* (Fig. 2d). Furthermore, we also confirmed the downregulation of *CsUGT74B5* in field-grown tea plants, specifically in the cultivars ‘Shuchazao’ and ‘Zhongcha 108’, consistent with laboratory findings, despite potential environmental interferences (Fig. S1). These results suggest that the glycosylation process catalyzed by CsUGT74B5 may be correlated with tea plants’ resistance to anthracnose.

### Expression profile of CsUGT74B5 in tea plants and subcellular localization

To investigate the transcription expression profile of *CsUGT74B5* across different tea plant organs, we performed qRT-PCR using cDNA templates from eight tea organs and tissues. The results showed that *CsUGT74B5* exhibited relatively high transcription levels in the third leaf, mature leaves, and stems, all of which have a high degree of maturity (Fig. S2a). These qRT-PCR results were consistent with the FPKM values obtained from public RNA-seq data [27] (Fig. S2b). In addition to anthracnose, we examined the response of *CsUGT74B5* to other biological stresses, including gray blight, and pests like *Ectropis oblique* and tea leafhopper (Fig. S2c). Public RNA-seq data revealed that while *CsUGT74B5* expression was not significantly affected by *E. oblique* and tea leafhopper (compared with mechanical damage), it was significantly downregulated during gray blight infection.

We also investigated the subcellular localization of *CsUGT74B5* in plants. A vector containing the expression cassette of 35S pro: *CsUGT74B5*-eGFP was transiently expressed in the leaves of *N. benthamiana.* Confocal microscopy revealed that the green fluorescence signals of *CsUGT74B5* were localized in the cytoplasm and nucleus, consistent with the localization patterns of the cytoplasmic and nuclear marker NF-YA4-mCherry (Fig. S2d).

### Phylogenetic analysis and functional prediction of CsUGT74B5

**Figure 3:**
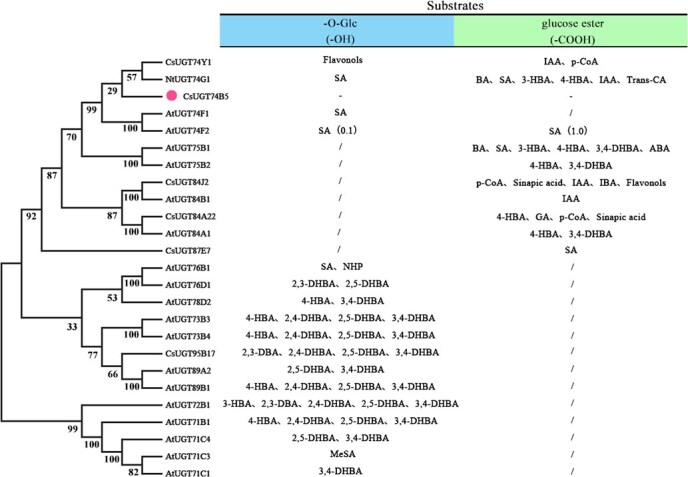
Phylogenetic and substrate prediction analysis of CsUGT74B5 with identified functional UGTs from other species. Left panel was the phylogenetic tree of CsUGT74B5 with the known-function UGTs from other species. Right panel listed the catalyzed substrates and the catalytic sites of these UGTs.

To predict the function of *CsUGT74B5*, we conducted a phylogenetic analysis comparing *CsUGT74B5* with other functionally characterized UGTs. The phylogenetic tree suggested that *CsUGT74B5* might possess catalytic activity toward benzoates (Fig. 3). Combing the list of substrates catalyzed by UGTs with known functions, CsUGT74B5 tended to catalyze the glycosylation at hydroxyl sites of substrates. Sequence alignment of *CsUGT74B5* with other reported UGTs revealed the presence of key substrate-binding sites, including sugar receptor and sugar donor binding sites (Fig. S3). The last amino acid of the conserved PSPG box is often indicative of sugar donor specificity (UDP–glucose or UDP–galactose) in plant glycosyltransferases [28]. Based on the last amino acid residue (Gln, Q) in the PSPG-Box of CsUGT74B5, it was speculated CsUGT74B5 might perform the catalytic function using UDP–glucose as a sugar donor.

### CsUGT74B5 specifically catalyzed the glycosylation of SA and 2,6-DHBA *in vitro*

**Figure 4:**
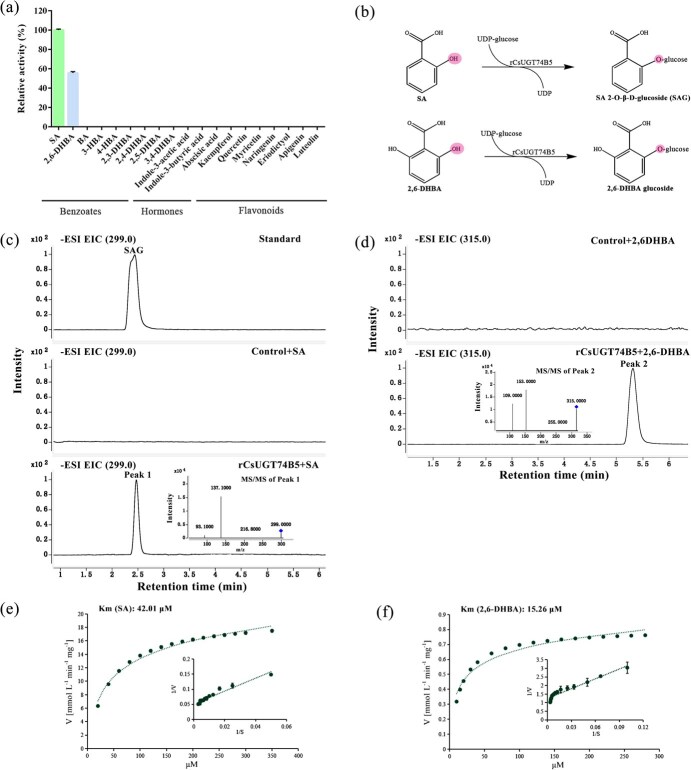
UPLC-MS analysis of the products in rCsUGT74B5-catalyzed reactions *in vitro*. (a) Activity screening of rCsUGT74B5 protein with various substrates. The catalytic activity for SA was set as 100%. Values are expressed as the mean ± standard deviation of triplicate samples. (b) The predicted glucosylation process and mechanism of SA and 2, 6-DHBA catalyzed by rCsUGT74B5. (c) UPLC-MS/MS analysis of the product in rCsUGT74B5-catalyzed reaction using SA as substrate. (d) UPLC-MS/MS analysis of the product of rCsUGT74B5-catalyzed reaction using 2,6-DHBA as the substrate. (e) Kinetic analysis of rCsUGT74B5 using SA as substrate. (f) Kinetic analysis of rCsUGT74B5 using 2,6-DHBA as substrate. All data presented here are the means of three replicates. Significant difference analysis (^*^P < 0.05, ^**^P < 0.01, ^***^P < 0.001.) was calculated by one-way ANOVA.

To verify whether CsUGT74B5 was involved in the biosynthesis of benzoates glycosides, the enzymatic activity of CsUGT74B5 was investigated with various benzoic acid derivatives as the acceptor substrates, and UDP-glucose served as donor substrate. Following protein induction, affinity purification, and SDS-PAGE test, the obtained recombinant CsUGT74B5 (rCsUGT74B5) protein had good quality and could be used for subsequent experiments (Fig. S4). The activities of recombinant CsUGT74B5 protein were first tested with benzoic acid derivatives as the substrate. It was shown that recombinant CsUGT74B5 could only catalyze the formation of SA glucoside and 2, 6-DHBA glucoside, exhibiting highest activity toward SA, followed by 2, 6-DHBA (Fig. 4a). The enzymatic activity of rCsUGT74B5 was further measured with other 10 acceptor substrates, including hormones and flavonoids. But no glucoside product was detected in any enzyme reaction.

The glucoside products of SA and 2, 6-DHBA were further identified through LC–MS. The SA glucoside (Peak 1) was identified as SAG by comparing with the SAG standard and the MS/MS ion fragments (Fig. 4b and c). The ions at m/z 299.0 and m/z 137.0 at negative MS spectra ([M-H]^−^) represented SAG and SA, respectively. The glucose of 2, 6-DHBA glucoside (m/z 315.0 at [M-H]^−^ mode) was identified to be attached to one of the hydroxyl groups of 2, 6-DHBA, the positions of which were symmetrical on the benzene. The ion fragment at m/z 153.0 represented 2, 6-DHBA, and ion at m/z 109.0 represented the resorcinol structure after being cracked (loss of carboxyl group) of 2, 6-DHBA at [M-H]^−^ mode (Fig. 4d). Likewise, the ion fragment at m/z 93.0 in Fig. 4c represented the phenol structure after being cracked of SA. These results indicated that CsUGT74B5 performed specific glycosylation activity at the *ortho* hydroxyl groups of benzoic acid derivatives. To determine the kinetic constants of rCsUGT74B5, the reaction conditions were firstly established and optimized, with UDP-glucose served as donor substrate and SA as the acceptor substrate. It was shown that the highest activity of rCsUGT74B5 was detected at pH 8.0–8.5 and 30°C (Figs S5 and S6). The kinetic constants of rCUGT74B5 were further investigated under optimal enzyme reaction conditions. The recombinant CUGT74B5 displayed higher affinity for 2,6-DHBA (*K*_M_ = 15.26 μM) (Fig. 4f), followed by SA (*K*_M_ = 42.01 μM) (Fig. 4e).

### Binding modes of various substrates with CsUGT74B5 generated by molecular docking

**Figure 5:**
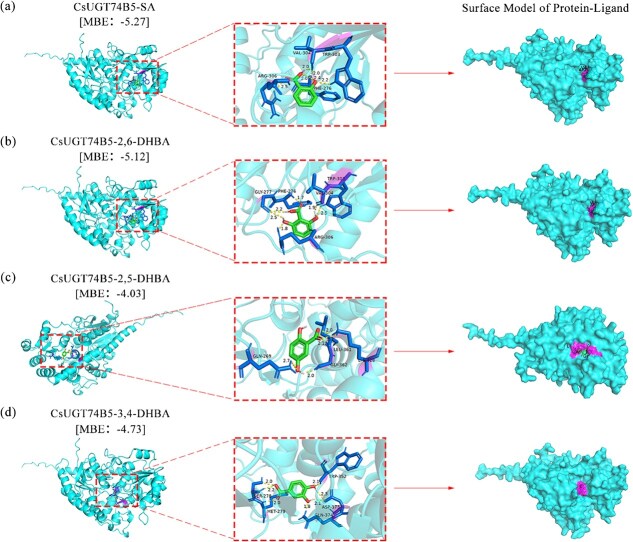
Molecular docking of CsUGT74B5 protein. (a) Molecular docking of SA binding to CsUGT74B5. (b) Molecular docking of 2,6-DHBA binding to CsUGT74B5. (c) Molecular docking of 2,5-DHBA binding to CsUGT74B5. (d) Molecular docking of 3,4-DHBA binding to CsUGT74B5 proteins. MBE represents the minimum binding energy. In the surface model of protein-ligand, the connection position of small molecules is displayed in purple.The CsUGT74B5 protein is displayed in blue, the small molecule ligand is displayed in green, the hydrogen bond is displayed in yellow, and the amino acid residues connected by hydrogen bonds in ligands are displayed in dark blue.

In order to further explain the catalytic characteristics and strict substrate selectivity of CsUGT74B5, molecular docking studies were performed. Four benzoic acid derivatives (SA, 2, 6-DHBA, 2, 5-DHBA, and 3,4-DHBA) were selected for molecular docking with predicted CsUGT74B5 protein structure based on the AlphaFold model. The binding postures for SA, 2, 6-DHBA, 2, 5-DHBA, and 3, 4-DHBA on CsUGT74B5 are shown in the Fig. 5. The minimum binding energy reflects the binding efficiency of the ligand to the receptor and the likelihood of an effect [29]. In molecular docking, a smaller value indicates better ligand binding. As shown in Fig. 5, these substrates and CsUGT74B5 protein structure displayed noncovalent interactions. The values of minimum binding energy of them were −5.27, −5.12, −4.03, and −4.73 kcal/mol, which indicated CsUGT74B5 retained higher binding ability to SA and 2, 6-DHBA than 2, 5-DHBA and 3, 4-DHBA. The surface model of molecular docking showed SA and 2,6-DHBA fully entered the binding cavity, 2,5-DHBA only partially entered the binding cavity, and 3,4-DHBA did not enter the binding cavity due to lack of hydrophobic interaction. In addition, the predicted binding sites of SA and 2,6-DHBA in CsUGT74B5 were basically consistent, including shared Phe-276, Trp-303, Val-304, and Arg-306 (Table S3). Summing up, the molecular docking analysis further supported our enzymatic activity results *in vitro*, that CsUGT74B5 was specifically glycosylating the ortho hydroxyl groups of benzoic acid derivatives.

### Heterologous expression of *CsUGT74B5* in *A. thaliana* and tobacco

**Figure 6:**
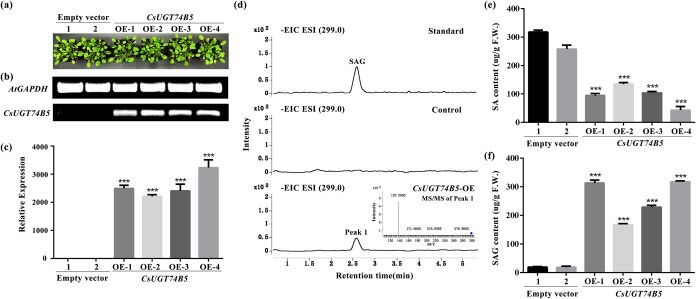
Overexpression of *CsUGT74B5* in *A. thaliana*. (a) Phenotype of empty vector and *CsUGT74B5*-overexpressing *A. thaliana* seedlings. (b) Semiquantitative RT-PCR analysis of *CsUGT74B5* and the housekeeping gene *AtGAPDH* in transgenic *A. thaliana*. (c) The relative expression levels of *CsUGT74B5* in different lines of transgenic *A. thaliana*. (d) Extraction ion chromatogram (EIC) of product SAG (m/z 299.0) accumulated in *CsUGT74B5* transgenic *A. thaliana*. The product (Peak 1) was further identified through comparison with authentic standards and MS/MS analysis. (e) The content of SA accumulated in control and *CsUGT74B5*-overexpressing *A. thaliana*. (f) The content of SAG accumulated in control and *CsUGT74B5*-overexpressing *A. thaliana*. All data presented are the means of three replicates. The error bars represent the standard deviation of three replicates. Asterisks indicate the significant level (*n* = 3, ^*^P < 0.05, ^**^P < 0.01, ^***^P < 0.001) based on a Tukey’s honestly significant difference test.

To verify the function of *CsUGT74B5* in plants, heterologous overexpression of *CsUGT74B5* in *A. thaliana* was performed. Multiple independent transgenic *A. thaliana* lines were successfully obtained, and overexpression of *CsUGT74B5* did not significantly alter the appearance phenotype compared to the control (Fig. 6a–c). Metabolite detection results showed the contents of SAG accumulated in different lines of *A. thaliana* overexpressing *CsUGT74B5* significantly increased compared with the control group (Fig. 6d and e). Due to the presence of only trace amounts of 2,6-DHBA, no 2,6-DHBA glycoside was detected in *CsUGT74B5* transgenic *A. thaliana* (data not shown).

**Figure 7:**
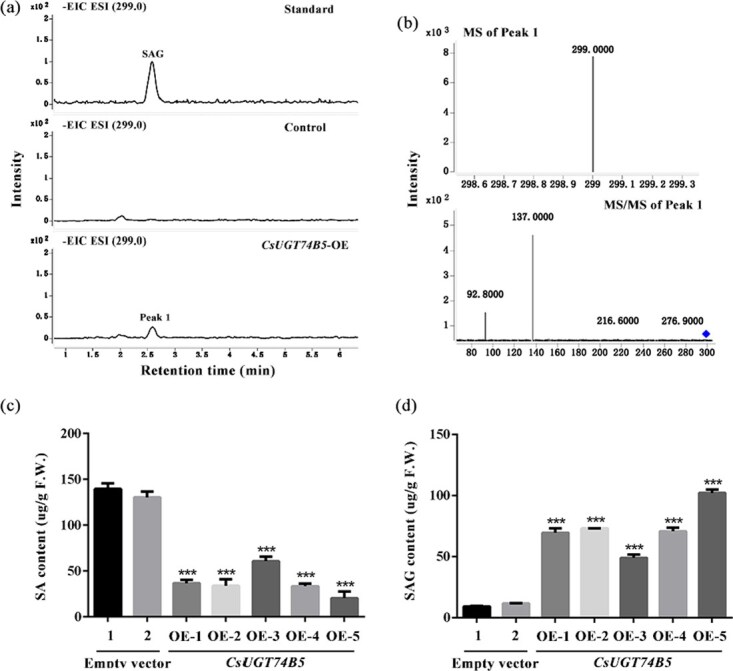
Metabolite analysis of products in *CsUGT74B5*-overexpressing tobacco. (a) EIC of product SAG (m/z 299.0) accumulated in *CsUGT74B5* transgenic tobacco using UPLC-MS. (b) UPLC-MS/MS analysis of the product (Peak 1, m/z 299.0) in transgenic *CsUGT74B5* tobacco. (c). The content of SA accumulated in control and *CsUGT74B5*-overexpressing tobacco. (d) The content of SAG accumulated in control and *CsUGT74B5*-overexpressing tobacco. All data presented here are the means of three replicates. Significant differences analysis of data (^*^P < 0.05, ^**^P < 0.01, ^***^P < 0.001) was calculated by one-way ANOVA.

Concurrently, *CsUGT74B5* was also overexpressed in the model plant tobacco. Similar to *A. thaliana*, overexpression of *CsUGT74B5* in tobacco significantly increased SAG accumulation, which correlated positively with *CsUGT74B5* transcription levels (Fig. 7a–c). Intriguingly, it was noted that the tobacco overexpressing *CsUGT74B5* was more susceptible to sulzer (*Myzus persicae*) infestation during cultivation, one kind of common insect in tobacco (Fig. S7). It was supposed that overexpression of *CsUGT74B5* in tobacco accelerated the transition from SA to SAG, resulting in a significant accumulation of SAG and a sharp decrease in SA content. These results indicated that CsUGT74B5 played a crucial role in converting SA to SAG in plants, fine-tuned the SA levels, and coordinated plant resistance.

### CsUGT74B5 modulates disease resistance in tea plants

**Figure 8:**
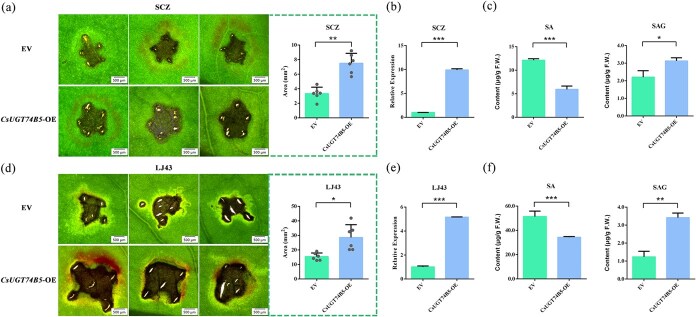
Effects of transient overexpression of *CsUGT74B5* on anthracnose infection on the tea leaves. (a, d) Comparison of lesion symptoms on the leaves of *CsUGT74B5*-overexpressing and control tea plants after inoculation of anthracnose strain at 5 dpi. ‘SCZ’ and ‘LJ43’ indicated tea cultivar ‘Shuchazao’ and ‘Longjing 43’, respectively. Scale bar = 0.5 mm. Error bars represent ± standard deviation (*n* = 6). (b, e) The relative expression level of *CsUGT74B45* after transient overexpression of *CsUGT74B5* using *Agrobacterium*-mediated transformation. (c, f) Detection of the contents of SA and SAG in the leaves of tea plants after overexpression of *CsUGT74B5*. All data presented here are the means of three replicates. Significant differences between the treatment and the control group were calculated by one-way ANOVA. ^*^P < 0.05, ^**^P < 0.01, ^***^P < 0.001.

Previous studies showed that anthracnose inhibited the expression level of *CsUGT74B5* in tea seedlings (Fig. 2). After verifying the function of CsUGT74B5 in glucosylating SA to SAG *in vitro*, the physiological role of CsUGT74B5 in tea plants needs to be further investigated. So, the effects of overexpression of *CsUGT74B5* on anthracnose infection on the tea leaves were tested (Fig. 8a and d). After transient overexpression of *CsUGT74B5*, the expression profile of CsUGT74B5 was determined at transcription and protein level, which showed CsUGT74B5 were significantly upregulated (Fig. 8b, Fig. S8). The result of inoculation experiment showed that tea leaves were more susceptible to anthracnose pathogen after transient overexpression of *CsUGT74B5* (Fig. 8). The lesion area on tea leaves overexpressing *CsUGT74B5* was significantly larger than that on tea leaves of the control group at 5 dpi, no matter in resistant cultivar ‘SCZ’ or susceptible cultivar ‘LJ43’ (Fig. 8a and d). UPLC-QQQ-MS analysis of metabolites showed that *CsUGT74B5*-overexpressing leaves had significantly reduced SA levels and markedly increased SAG levels compared to the control group (Fig. 8c and f). These results demonstrate that overexpression of *CsUGT74B5* in tea plants promotes SAG biosynthesis, leading to reduced SA levels and increased susceptibility to anthracnose. Thus, *CsUGT74B5* negatively modulates anthracnose resistance in tea plants by reducing SA levels.

### Exogenous SA improved the tolerance of tea plants to anthracnose

**Figure 9:**
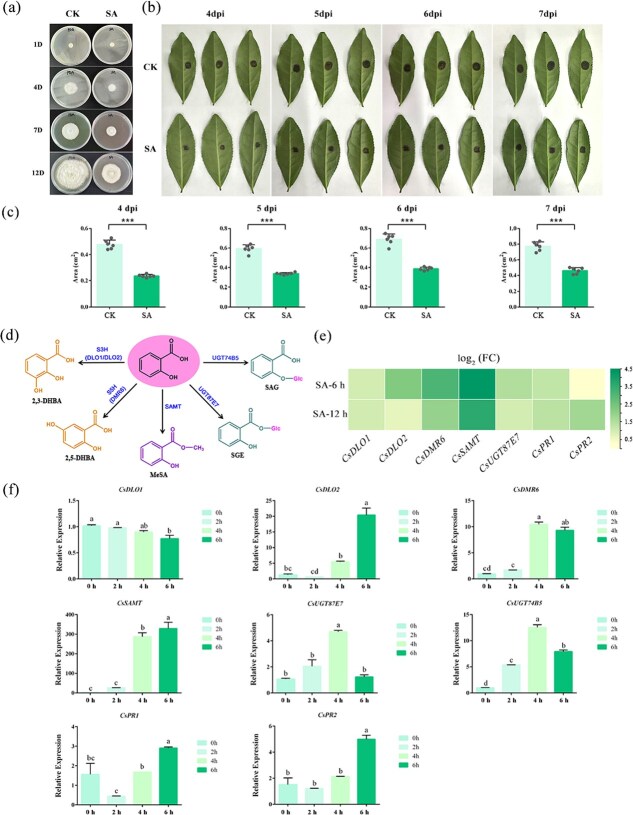
The inhibitory effects of exogenous SA on the infection of anthracnose pathogen. (a) Anthracnose was inoculated on PDA medium supplied with 1 mM SA. (b) Comparison of lesion symptoms on the leaves after SA application. (c) The lesion area value statistics on the leaves after SA application. Error bars represent ± standard deviation (*n* = 6); (d) SA downstream metabolic pathway; (e) Heat map of the ratio of FPKM values (SA-6 h/CK-6 h and SA-12 h/CK-12 h) in SA downstream pathway based on RNA-seq data. (f) The qRT-PCR results of genes in SA downstream pathway after SA treatment for 2, 4, and 6 h. All data presented here are the mean ± SE of three replicates.

Following anthracnose infection, SA levels in tea leaves significantly increased, and reduced SA levels were associated with increased susceptibility to anthracnose (Figs 2 and 8). However, it was unclear whether exogenous SA application could affect anthracnose infection. Therefore, we conducted *in vitro* assays for antifungal activity and sprayed tea seedlings with SA after anthracnose inoculation. As shown in Fig. 9a, mycelial growth of the anthracnose strain was significantly inhibited on the media with 1 mM SA. Similarly, it was observed that anthracnose lesions spread more slowly on SA-treated leaves (Fig. 9b). The lesion area values on the leaves sprayed with SA were significantly lower than those of their corresponding control groups at 4, 5, 6, and 7 dpi (Fig. 9c). Therefore, it was concluded that SA played a critical role in resisting the occurrence of anthracnose *in vitro* and in tea plants.

Additionally, we examined the expression levels of genes involved in the SA downstream metabolic pathway and pathogen-related (PR) genes in tea seedlings treated with exogenous SA (Fig. 9d). RNA-seq data of SA-treated tea seedlings (SA-6 h and SA-12 h) showed that exogenous SA treatment upregulated the expression levels of SA-related genes, including *CsS3Hs* (*DLO1*, CSS0021865; *DLO2*, CSS0030853), *CsS5Hs* (*S5H1*, CSS0032224; *S5H2*, CSS0044637), *CsSAMT* (CSS0035688), *CsUGT87E7* (C*SS000206*6), and *CsPR1* (CSS0007078), particularly at SA-6 h stage (Fig. 9e) [30]. Consistent with RNA-seq data, qRT-PCR results showed that the expression levels of these genes, except *CsDLO1*, were upregulated after 2, 4, and 6 h of SA treatment (Fig. 9f). The qRT-PCR results revealed the transcription level of *CsUGT74B5* was upregulated by 5- to 10-fold after short-term (2–6 h) SA treatment. The expression levels of *CsPR1* (CSS0007078) and *CsPR2* (CSS0000190) were also significantly upregulated, potentially aiding tea plants in coping with anthracnose infection.

## Discussion

### The regioselectivity of CsUGT74B5 towards the substrates

Glycosylation is an essential step in the secondary metabolism of plants, comprising phenolics, terpenoids, alkaloids, and other compound metabolism. Plant glycosyltransferases not only possess multisubstrate catalytic activity, but often exhibit regioselectivity during the catalytic reaction process *in vitro*. Two forms of glycosylated products of SA accumulated in plants, one is SAG and the other is GS. AtUGT74F1 and AtUGT74F2 catalyzed the synthesis of them in *Arabidopsis*, respectively. Interestingly, while AtUGT74F2 can catalyze the formation of small amounts of SAG, it exhibits more stringent regioselectivity, favoring carboxyl glycosylation despite its high homology with AtUGT74F1. In tea plants, CsUGT87E7 is responsible for SA carboxyl glycosylation, while CsUGT74B5 is responsible for SA hydroxyl glycosylation. But unlike in *Arabidopsis*, CsUGT87E7 shares lower homology with CsUGT74B5. CsUGT74B5 displayed highest activity toward SA and 2, 6-DHBA, glucosylating at the *ortho* hydroxyl groups of the substrates, but with zero activity toward 2, 3-DHBA, 2, 4-DHBA, 2, 5- DHBA, and 3, 4-DHBA. The phenomenon was not surprising, as glycosylation activity at specific sites could be negatively or positively influenced by the presence of additional hydroxyl groups on the benzene ring. For instance, AtUGT75B1 catalyzed the glycosylation at the carboxyl group of the aglycone, forming a glucose ester, but the activity of AtUGT75B1 toward the carboxyl group on the benzoate compounds was inhibited when the substrate carries a 2- or 2, 6-hydroxyl group(s) [1]. The molecular docking result showed SA and 2,6-DHBA fully entered the binding cavity, 2, 5-DHBA only partially entered the binding cavity, and 3, 4-DHBA did not enter the binding cavity (Fig. 5). The additional hydroxyl groups at 3-OH, 4-OH, and 5-OH of the benzene ring might limit or obstruct the entry of substrates into the catalytic pocket, thereby decreasing the substrate-binding ability with the catalytic sites.

Therefore, CsUGT74B5 was a unique glucosyltransferase, transferring the glucose to the *ortho* hydroxyl groups of benzoic acid derivatives. Currently, there are no reports of UGT glycosylating 2, 6-DHBA into corresponding glycosides; CsUGT74B5 will fill this gap in the field of biochemistry. Identification of the key amino acid residues involved in catalyzing the ortho hydroxyl glycosylation of benzoic acid needs to be further studied, which will provide reference information for identifying the functionality of UGTs. The roles of CsUGT74B5 in tea plants will be further explored and discussed.

### Diversified metabolic pathways fine-tuned SA homeostasis and pathogen resistance

**Figure 10:**
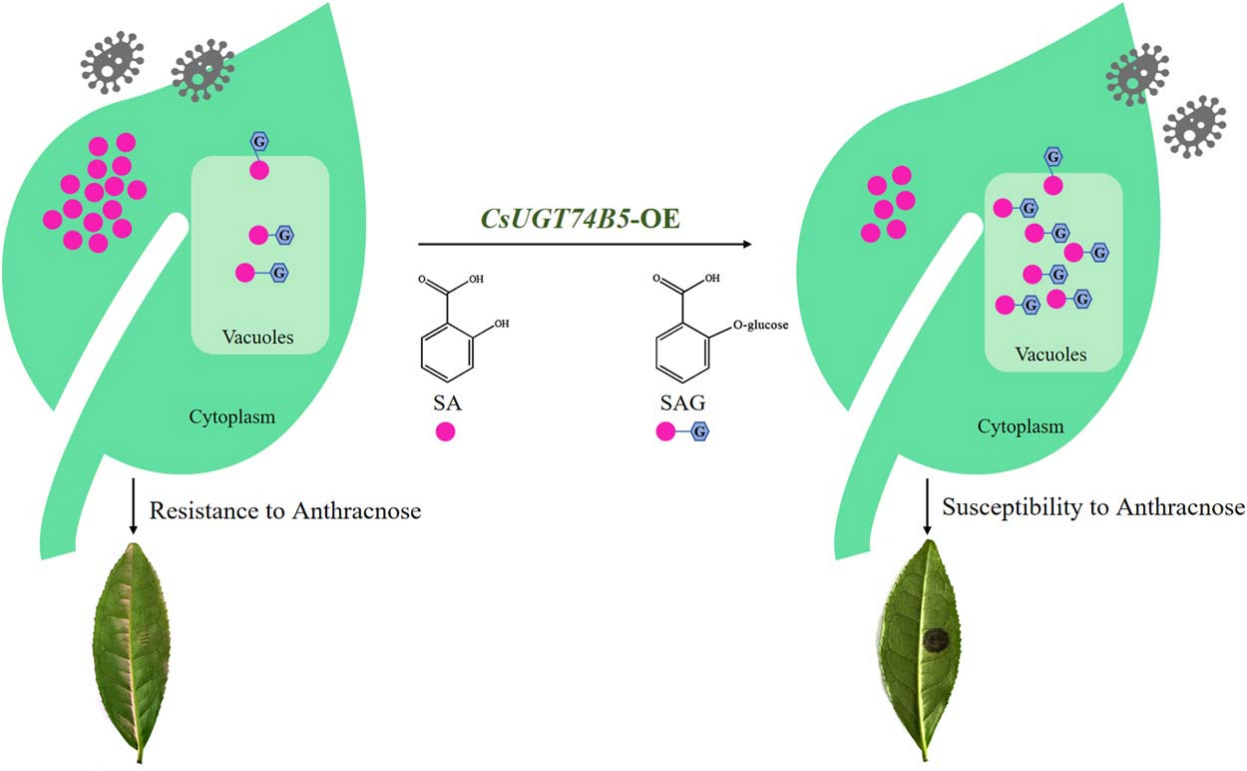
Mechanism model of CsUGT74B5 function in fine-tuning the SA levels and coordinated plant resistance to anthracnose

To confer disease resistance to plants, two strategies are often adopted. One strategy is the screening and deployment of single resistance genes, which is the common means of conventional breeding. Another strategy is disabling one or more susceptibility genes in plants, weakening the perception of plants to pathogens. However, the former strategy has been easily overcome by constantly evolving pathogens. Compared with screening resistance genes, disabling susceptibility genes is a more promising breeding program, as it can offer durable and broad-spectrum disease resistance. Benzoic acid derivatives are closely related to plant disease resistance, such as SA, 3,4-DHBA, and the glycosides of 2,4-DHBA, 2,5-DHBA, and SA [4, 21, 22, 26]. SA, as the most common plant resistance signal and hormone, plays an important role in plant resistance to external pathogens. In tomato and rice, silencing of a susceptibility factor (DMR6, which performs SA hydroxylation function) enhanced plant mutants’ resistance to different classes of pathogens, and the disease resistance was closely related to increased SA levels [14, 16, 31]. In our study, recombinant CsUGT74B5 catalyzed glycosylation at the *ortho* hydroxyl groups of SA and 2, 6-DHBA *in vitro*, generating SAG and 2, 6-DHBA glycoside. Consistent with this, overexpression of *CsUGT74B5* in tea plants and tobacco increased their sensitivity to anthracnose and insect attacks, respectively. It was due to the overexpression of *CsUGT74B5* in plants promoting the SA glycosylation and reduced the content of free SA. So, a mechanism model of CsUGT74B5 function in fine-tuning the SA levels and coordinating plant resistance to anthracnose in tea plants was proposed (Fig. 10). Different from the catalytic mechanism of S5Hs (also named DMR6), which degraded SA into dihydroxybenzoic acids through SA hydroxylation at 3- or 5-hydroxyl group(s), SA glycosylation catalyzed by CsUGT74B5 mainly performed the function of transferring and storing SA in another form (SAG).

SA downstream metabolism consisted of several catabolic pathways, including hydroxylation, glycosylation, methylation, and others. GS, another glycosylated product of SA, is catalyzed by AtUGT72F2 in *Arabidopsis* [19]. In tea plants, a UGT (CsUGT87E7) induced by tea gray blight could glycosylate SA to form GS, which played a crucial role in modulating tea plant disease resistance [26]. Currently, there are few reports on the involvement of SA hydroxylation in SA metabolism and plant immune responses in tea plants. Moreover, the dominance of specific catabolic pathways in SA metabolism remains unclear in plants. It is also unknown whether the metabolic flow of SA is related to the type of exogenous disease or pest, which is a topic we will explore further in future studies.

## Materials and methods

### Plant materials and growing conditions

Two-year-old tea plant cultivars ‘Shuchazao (SCZ)’ and ‘Longjing43 (LJ43)’ were obtained from the experimental tea garden of Qingdao Agricultural University. The tea seedlings were acclimatized in a growth chamber for 2 weeks before undergoing various treatments. The *A. thaliana* material was ecotype Columbia 0 (Col-0), which was grown in a constant temperature of 16 ± 2°C with a 16 h (light)/8 h (dark) photoperiod. Tobacco plants were cultivated in a growth chamber at 25 ± 2°C with a 12/12 h (light/dark) photoperiod.

### Gene cloning and qRT-PCR analysis

The open reading frame (ORF) of *CsUGT74B5* was amplified using Phusion™ High-Fidelity DNA Polymerase (Thermo Scientiffc™), then the polymerase chain reaction (PCR) products were cloned into the pEASY-Blunt cloning vector and subsequently transformed into Trans-T1 competent cells for sequencing. The primers used for gene cloning and vector construction are listed in Table S1. Quantitative reverse transcription-polymerase chain reaction (qRT-PCR) procedure was performed following the previously reported method [30]. All experiments were conducted in triplicate. The relative expression level was calculated following a previously published method (2^–ΔΔCT^ computing method), using *CsGAPDH* as an internal reference gene [32]. The primers used in qRT-PCR are listed in Table S2.

### Prokaryotic expression of CsUGT74B5 and protein purification

The CDS sequence of *CsUGT74B5* was inserted into the protein expression vector pMAL-c2X. After sequencing, the pMAL-*CsUGT74B5* vector was transformed into *Escherichia coli* strain Novablue (DE3) for protein expression. When the OD_600_ of the transformed cells reached 0.6–0.8, protein expression was induced with 1 mM IPTG at 28°C for 16 h. The recombinant CsUGT74B5 protein was purified by affinity chromatography using amylose resin (New England Biolabs, MA, USA), following the method described in Dai’s research [33]. Protein concentration was determined photometrically, and a 12% sodium dodecyl sulphate-polyacrylamide gel was used to assess the quality of purified CsUGT74B5 protein. The purified protein was then used for *in vitro* enzymatic assays.

### Anthracnose inoculation in tea plants

The anthracnose strain used in our study was isolated from tea plant cultivar ‘Taoyuandaye (TYDY)’ in Hunan province, named ‘TYDY-2’. The pathogenic isolate (TYDY-2), identified as *C. camelliae* and highly virulent to tea plants, was used for anthracnose inoculation [34]. The anthracnose strain was cultured on potato dextrose agar (PDA) medium at 28°C for 4–5 days. Tea seedling leaves were disinfected with 75% alcohol, and five puncture wounds were made on the abaxial side of the leaves using a syringe needle. A circular fungal block (0.8 cm in diameter) was then applied to each wound. The blocks were covered with plastic wrap to prevent drying. After 48 h, the fungal blocks were removed, and the tea seedlings were incubated at 70% humidity. Leaf samples surrounding the infected lesions were collected on days 3, 4, 5, 6, 7, and 9 postinoculation, then immediately frozen in liquid nitrogen for subsequent RNA isolation and metabolic analysis. The tea leaves only being disinfected with 75% alcohol and wounded (without fungal block) were used as the mock-inoculated leaf samples.

### Enzymatic activity assays

Enzymatic assays and kinetic analyses of the recombinant rCsUGT74B5 protein were conducted in 50-μl reaction mixtures containing 20 μg of purified protein, 2.5 mM UDP-glucose (UDPG), and 200 μM substrates, with the volume adjusted to 50 μl using 100 mM Tris–HCl (pH 7.4). All the enzyme reactions were initiated by adding the purified protein and incubated for 30 min at 30°C. Enzyme reactions were terminated by adding 50 μl of methanol, and 50 μl of the reaction mixture was injected for HPLC analysis.

To determine the optimal reaction temperature, enzyme reactions (pH 7.4, 100 mM Tris–HCl) were incubated at temperatures ranging from 0°C to 65°C in 5°C increments. Optimal reaction pH was tested using 100-mM buffers: citric acid-sodium citrate (pH 4.0–6.0), Tris–HCl (pH 6.0–9.0), phosphate (pH 6.0–9.0), or Na_2_CO_3_/NaHCO_3_ (pH 9.0–11.0) with 0.5-pH increments, incubated at 30°C. All reactions lasted 45 min and were terminated with 50 μl of chromatographic methanol.

For kinetic parameters of rCsUGT74B5, substrate concentrations ranging from 0 to 300 μM, including SA and 2,6-DHBA, were used under optimal conditions with 2.5 mM UDP-glucose as the sugar donor. All the enzyme reactions lasted 30 min and performed in triplicate. After being terminated with 50 μl of chromatographic methanol, samples were stored at −80°C before liquid chromatography-mass spectrometry (LC-MS) analysis.

### *Agrobacterium*-mediated transient overexpression of *CsUGT74B5* in tea plants

The ORF of *CsUGT74B5* was cloned into the pBTEX-1036-HA vector and transferred into *Agrobacterium tumefaciens* GV3101. After confirmation by PCR, the GV3101 strain containing the target construct was cultured in LB medium until the OD_600_ reached 0.6. The cells were collected by centrifugation, washed, and resuspended in MES suspension following previously reported method [25]. The bacterial suspension was injected into the abaxial side of mature tea leaves using a 0.5-ml needle-free syringe until the entire leaf was infiltrated. GV3101 strains carrying the empty vector pBTEX-1036-HA were used as the control. After 48 h in the dark, the tea seedlings were returned to normal growth conditions. Leaf samples were collected at 2, 3, 4, and 5 days postinfection, then immediately frozen in liquid nitrogen for subsequent RNA isolation and metabolic analysis. Primers for binary vector construction are listed in Table S1.

### Western blot assay

Two hundred milligrams of fresh tea leaf samples were ground into powder in liquid nitrogen and collected into a 2.0-ml centrifuge tube. Then 1.0 ml protein extraction solution (95 mM 3- (N-Matholino) propanesultonic acid (MOPS), 150 mM NaCl, 20 mM 2-Iodoacetamide, 1% Triton X-100) was added into this tube. The tubes were vortexed for 30 s for protein dissolution. The samples were then centrifuged at 13 000 rpm for 20 min, and the supernatant was pipetted into a new tube for protein concentration measurement. The plant protein samples (20 μg) were separated through 12% sodium dodecyl sulfate-polyacrylamide gel electrophoresis (SDS-PAGE), followed by transfer onto polyvinylidene fluoride (PVDF) membranes. The parameters of the apparatus were set as 300 mA for 2 h. After that, the PVDF membranes were firstly sealed for 2 h with 5% skimmed milk powder, followed by being incubated with antibody (1:10 000) overnight at 4°C. Then the PVDF membranes were incubated with secondary antibody (1:10 000) at room temperature for 3 h. Finally, the membranes were fully washed and colored by ECL method, before being observed by a plant live fluorescence detector. The β-actin protein was used as the internal control in this assay.

### Anthracnose inoculation on the tea seedlings overexpressing *CsUGT74B5*

Transient overexpression of *CsUGT74B5* in tea seedlings was performed as described above, and ~25 tea seedlings overexpressing *CsUGT74B5* were obtained. After verifying the upregulation of *CsUGT74B5* expression via qRT-PCR, the leaves were subjected to anthracnose inoculation. Lesions were observed and recorded daily. Leaf samples surrounding the infected lesions were collected and immediately frozen in liquid nitrogen for subsequent RNA isolation and metabolic analysis.

### Heterologous expression of *CsUGT74B5* in *A. thaliana* and tobacco

The ORF of *CsUGT74B5* was first cloned into the entry vector pDONR207 using the Gateway BP Enzyme mix (Invitrogen) following the instructions. After sequencing, the pDONR207-*CsUGT74B5* vector was then transferred to the destination vector pCB2004 using LR Enzyme mix (Invitrogen). The expression vector with the target gene (pCB2004-*CsUGT74B5*) was then transformed into *A. tumefaciens* strain GV3101. Primers for binary vector construction are listed in Table S1. The *A. tumefaciens* cells were transferred into *A. thaliana* and tobacco using the floral-dip and leaf disk protocols, respectively, as previously described [35, 36]. The transgenic *A. thaliana* and tobacco lines with higher transcription levels, verified by semi-qRT-PCR and qRT-PCR, were selected for further study. The transgenic *A. thaliana* and tobacco used in our study were homozygous T3 generation and T2 generation, respectively.

### Extraction and quantification of SA and SA glucosides

For metabolite analysis, plant samples were ground in liquid nitrogen. Seventy-five milligrams of each sample was extracted with 1 ml ethyl acetate. The solutions were vortexed, sonicated at 4°C for 15 min, and centrifuged at 12 000 rpm at 4°C for 15 min. The supernatant was transferred to a new 2-ml centrifuge tube and evaporated under nitrogen. Two hundred μl of 50% methanol was added to the evaporated tube, followed by sonication for 15 min. The solution was centrifuged at 13 000 rpm at 4°C for 20 min, and the supernatant was transferred to a 1.5-ml centrifuge tube. All the samples were filtered through a 0.22-μm membrane prior to ultra-performance liquid chromatography-mass spectrometry (UPL-MS) analysis.

Ultra-performance liquid chromatography-tandem quadrupole mass spectrometry (UPLC-QQQ-MS) (Agilent) was used to identify and quantify SA and SAG. Multiple reaction monitoring (MRM) mode in LC–MS was developed for quantification of SA and SAG following previous reported protocol [37]. A C18 column was used to separate our target’s metabolites. The mobile phase in our experiment was 0.4% acetic acid (eluent A) and 100% acetonitrile (eluent B). Electrospray ionization (ESI) was used to acquire the mass spectra (from 100 to 1000) of metabolites in negative mode. The drying gas temperature, drying gas flow, nebulizer pressure, and capillary voltages were set at 350°C, 6 l/min, 45 psi, and 3500 V, respectively. The ion scanning range was set from m/z 100 to m/z 1000 and the fragmentation voltage was set at 175 V.

### Subcellular localization

The *CsUGT74B5*-pCAMBIA1300 vector was introduced into *A. tumefaciens* GV3101 by chemical transformation. A positive colony was selected and cultured before injecting the leaves of *Nicotiana benthamiana* using a previously reported protocol. After 72 h, the leaves were examined using a confocal microscope (TCS SP8; Leica, Wetzlar, Germany). Plasma membranes and nuclei were visualized by cotransformation with mCherry-labeled plasma membrane marker (PM-mCherry) and mCherry-labeled nuclear marker (NF-YA4-mCherry). For green fluorescence observation, the excitation wavelength was set to 488 nm with emission at 520–540 nm; for red fluorescence, the excitation wavelength was set to 561 nm with emission at 610–630 nm. Confocal microscope settings were kept consistent for comparing nuclear and cytoplasmic signals.

### Molecular docking

Molecular docking simulation was performed using AutoDock 4.2.6 software, and the binding energy was calculated with AutoDockTools. The SDF files of ligands (four kinds of benzoic acid derivatives) were downloaded from Pubchem (https://pubchem.ncbi.nlm.nih.gov). Using AutoDock 4.2.6 software, the structure of CsUGT74B5 protein model was optimized for dehydration, hydrogenation, and removal of unreasonable heteroatoms. Then the ligand molecules were also hydrogenated, checked for torsion centers, and docked with CsUGT74B5 protein model using AutoDock 4.2.6 software. Semiflexible docking was adopted in our molecular docking study, allowing for some changes in the molecular conformation during the docking process, which not only improved the accuracy but ensured the computational efficiency. Based on the binding site provided by molecular docking, use AutoDockTools tool to set the location of ligands on receptor and other parameters to obtain DLG files. Then the DLG files were analyzed through AutoDockTools and transformed into PDB files using OpenBabel software. Afterwards, the PDB file was uploaded to the PLIP website (https://projects.biotec.tu-dresden.de/plip-web/plip) for analyzing the binding mode between ligands and proteins. The molecular docking of ligands and receptor were finally visualized using Pymol software.

### *In vitro* assays for antifungal activity

A circular fungal block (0.8 cm in diameter) of the anthracnose strain (*C. camelliae*) was inoculated on the PDA medium center containing 1 mM SA and cultivated at 28°C. Hyphal inhibition was checked and the hyphal growth was observed and photographed daily. The *in vitro* antifungal activity assays were performed as described by Yuan et al [38].

### SA treatment on tea seedlings

Two hundred tea seedlings were selected for SA treatment. All the leaves of tea plants were sprayed with 5 mM SA following our previous study [30]. Then tea samples were collected at 0 (before spraying), 2, 4, and 6 h post-treatment, then immediately frozen in liquid nitrogen for RNA isolation. Three independent biological replicates were harvested for each sample to minimize biological variance.

### Inhibition experiment of exogenous SA on anthracnose pathogen

Anthracnose inoculation on the tea leaves was performed as described in Section 2.4. After removing the fungal blocks, tea seedling leaves were evenly sprayed with 1 mM SA, which was predissolved in 0.005% silwet aqueous solution. The control leaves were sprayed with an equal volume of 0.005% silwet aqueous solution. The degree of infection on the leaves was observed and photographed daily.

### Construction of phylogenetic tree

The phylogenetic tree was constructed by neighborhood-connection method with a bootstrap value of 1000, using MEGA 11 software. The evolutionary distances were calculated using the JTT matrix-based method and were in the units of the number of amino acid substitutions per site. The phylogenetic tree analysis involved 25 amino acid sequences. All ambiguous positions were removed for each sequence pair.

### Statistical analysis

All data were analyzed in triplicate, except for Fig. 7d (*n* = 5) and 8a, 8d (*n* = 6), 9b (*n* = 6). The results are presented as mean values with corresponding standard deviations. SPSS software (version 21.0) was used for variance analysis. Duncan’s multiple range test and Student’s *t*-test were used to evaluate significant differences at *P* < 0.05 (*), *P* < 0.01 (**), and *P* < 0.001 (***).

## Supplementary Material

Web_Material_uhaf009

## Data Availability

The data that support the findings of this manuscript are available in the article and the supplementary Tables and Figures. The transcriptome data had been uploaded to the Sequence Read Archive database on NCBI with the accession number of PRJNA857833.
