# Supplementary material for: UGT74B5-mediated glucosylation at ortho hydroxyl groups of benzoic acid derivatives regulating plant immunity to anthracnose in tea plants
Source: Hortic Res. 2025 Jan 14;12(4):uhaf009. doi: 10.1093/hr/uhaf009 (PMC11908824; doi:10.1093/hr/uhaf009)
Supplement: Web_Material_uhaf009 [file web_material_uhaf009.zip › Supplementary Figs .docx]

**The Supplementary Figures of**

**UGT74B5-Mediated Glucosylation at *ortho* Hydroxyl Groups of Benzoic Acid Derivatives Regulating Plant Immunity to Anthracnose in Tea Plants**

Caiyun Li ^1§^, Feixue Wu ^1§^, Nana Liu ^1^, Lei Yang ^1^, Xinfu Zhang ^1^, Fengfeng Qu ^1^, Liping Gao ^3^, Tao Xia ^2^, Lei Zhao ^1*^ Peiqiang Wang ^1*^

**Figure. S1** The relative expression levels of *CsUGT74B5* in the leaves of tea plants infected with anthracnose;

**Figure. S2** The expression profile of CsUGT74B5 in tea plants and subcellular localization

**Figure. S3** Sequence alignment of CsUGT74B5 with other reported UGTs

**Figure. S4** SDS-PAGE analysis of recombinant CsUGT74B5 expressed in *E. coli*.

**Figure. S5** Effect of reaction pH on the activity of rCsUGT74B5. Data are presented as mean ± SE of at least three repetitions.

**Figure. S6** Optimization of the reaction temperature of rCsUGT74B5 in vitro. Data are presented as mean ± SE of at least three repetitions.

**Figure. S7** Comparison of symptoms and quantity statistics of sulzer (*Myzus persicae*) in control and transgenic CsUGT74B5 tobacco.

**Figure. S8** Analysis of CsUGT74B5 protein expression levels in tea leaves after transient overexpression determined by western blot.


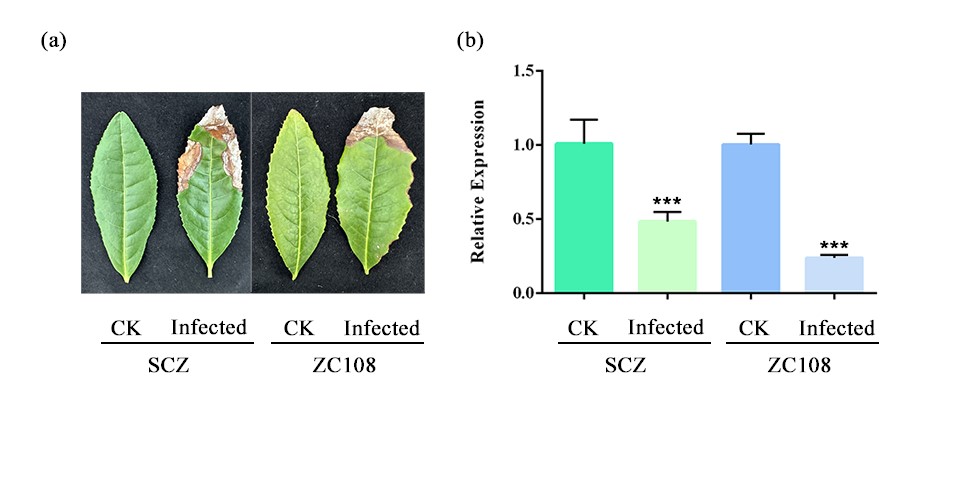


**Figure. S1** The relative expression levels of *CsUGT74B5* in the leaves of tea plants infected with anthracnose.

(a) The symptoms on the leaves of tea plants infected with anthracnose. Two tea cultivars in the tea garden were observed and photographed for record; ‘SCZ’ indicated the cultivar ‘Shuchazao’, ‘ZC108’ indicated the cultivar ‘Zhongcha 108’. (b) The relative expression levels of *CsUGT74B5* in the leaves of tea plants infected and uninfected with anthracnose in the tea garden.

**
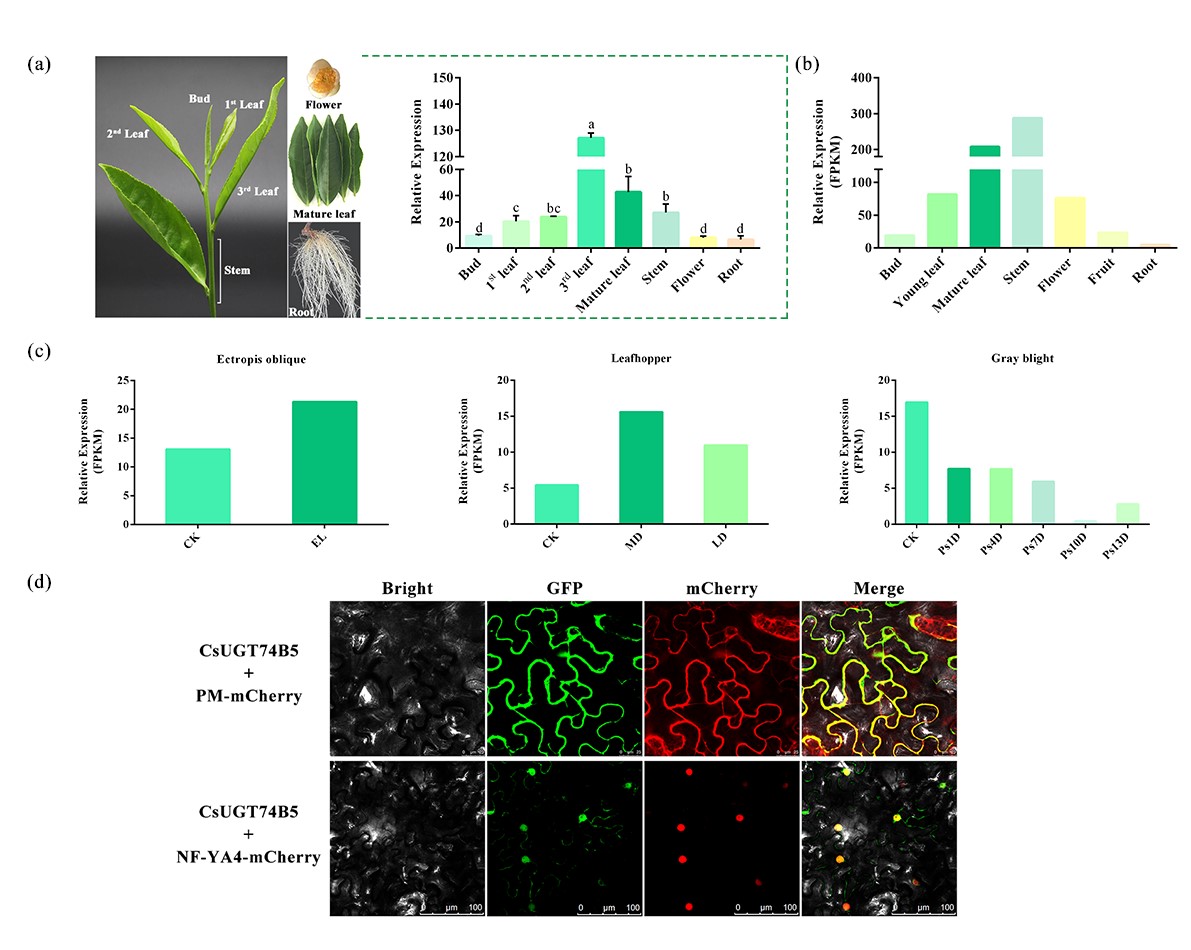
**

**Figure. S2.** The expression profile of CsUGT74B5 in tea plants and subcellular localization

(a) The relative expression level of CsUGT74B5 in eight tissues of tea plants determined by qRT-PCR; Different letters on the columns indicated the significantly different analysis at P < 0.05. (b) The relative expression level of CsUGT74B5 in seven tissues of tea plants reflected by RNA-sequencing. Data obtained from a public transcriptome database (TPIA; <http://tpia.teaplant.org>). MD, mechanical damage. (c) The relative expression level of CsUGT74B5 in tea plants under three biological stresses reflected by RNA-sequencing. Data were obtained from the tea public transcriptome database (TSA: PRJNA343349). (d) Subcellular localization of CsUGT74B5 in Nicotiana benthamiana. GFP was fused to the C-terminus of these proteins. The distribution of the green fluorescence signals indicated the location of CsUGT74B5 protein in plants. PM-mCherry and NF-YA4-mCherry are the positive cytoplasm marker and nuclear localization marker, respectively.


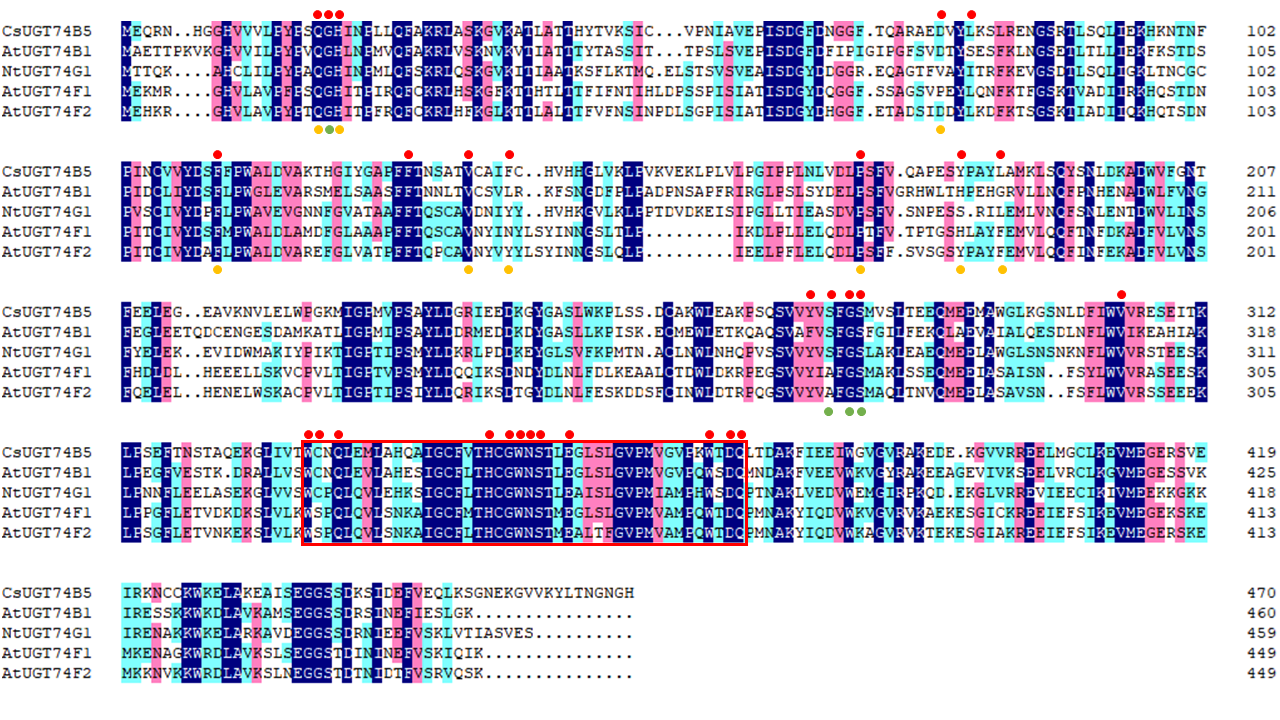


**Figure. S3** Sequence alignment of CsUGT74B5 with other reported UGTs

Red box: Conserved sequence of UGTs gene family (PSPG box); Red dots: catalytic sites; Yellow dots: sugar receptor binding sites; Green dots: sugar donor binding sites.


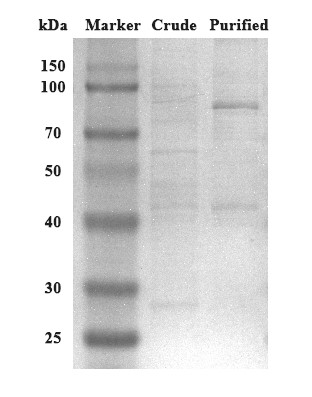


**Figure. S4** SDS-PAGE analysis of recombinant CsUGT74B5 expressed in *E. coli*.


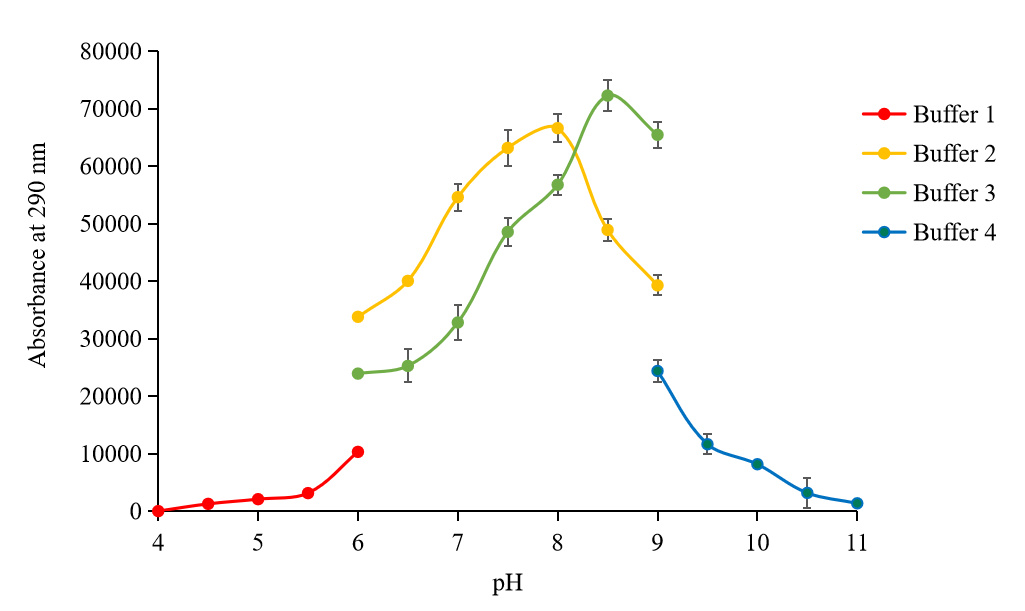


**Figure. S5** Effect of reaction pH on the activity of rCsUGT74B5. Data are presented as mean ±SE of at least three repetitions.

**
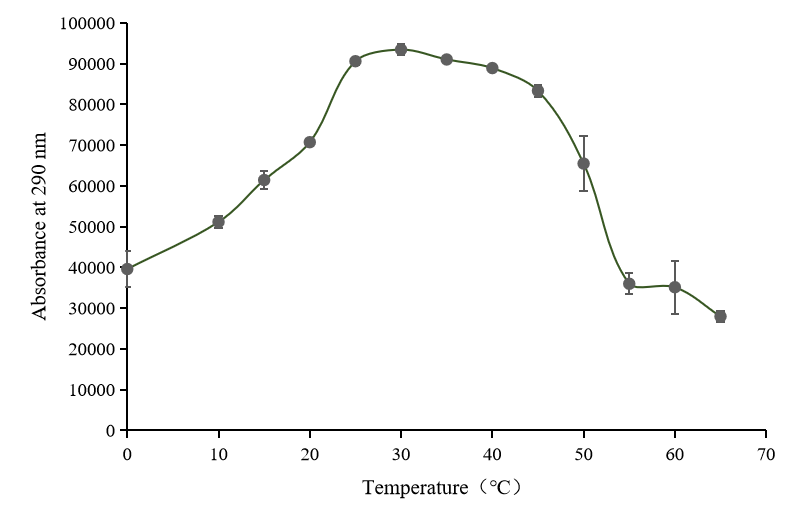
**

**Figure. S6** Optimization of the reaction temperature of rCsUGT74B5 in vitro. Data are presented as mean ± SE of at least three repetitions.


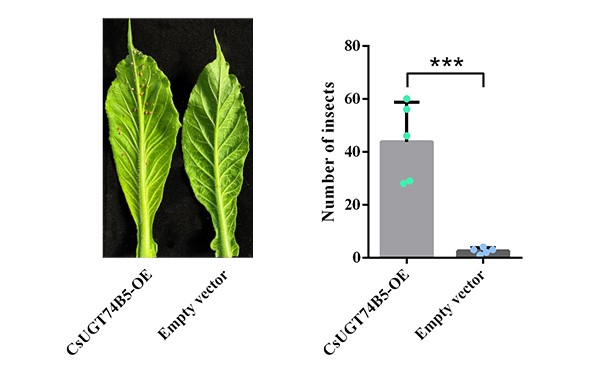


**Figure. S7** Comparison of symptoms and quantity statistics of sulzer (Myzus persicae) in control and transgenic CsUGT74B5 tobacco. Error bars represent ± standard deviation (n = 5).


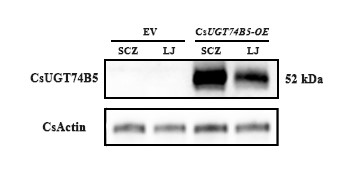


**Figure. S8** Analysis of CsUGT74B5 protein expression levels in tea leaves after transient overexpression determined by western blot.

EV represents empty vector control. ‘SCZ’ and ‘LJ’ indicated tea cultivar ‘Shuchazao’ and ‘Longjing 43’, respectively.
