# Supplementary material for: UGT74B5-mediated glucosylation at ortho hydroxyl groups of benzoic acid derivatives regulating plant immunity to anthracnose in tea plants
Source: Hortic Res. 2025 Jan 14;12(4):uhaf009. doi: 10.1093/hr/uhaf009 (PMC11908824; doi:10.1093/hr/uhaf009)
Supplement: Web_Material_uhaf009 [file web_material_uhaf009.zip › Supplementary Tables .docx]

**Table S1.** Primers used for cloning Cs*UGT74B5* and vector construction.

| Purpose | Primer name | Primer sequence (5'-3') |
| --- | --- | --- |
| DNA cloning | *CsUGT74B*5-F | ATGGAGCAAAGAAACCATGG |
|  | *CsUGT74B5*-R | TCAATAATGACCATTTCCATTGG |
| Construction of protein expression vectors | *CsUGT74B5-Pmal*-F | GAAGGATTTCAGAATTCGGATCCATGGAGCAAAGAAACCATGG |
|  | *CsUGT74B5-Pmal*-R | CTGCAGGTCGACTCTAGAGGATCCTCAATAATGACCATTTCCATTGG |
| Construction of  overexpression vectors | *CSUGT74B5-PBTEX*-F | ATTTGGAGAGGACAGGGTACCATGGAGCAAAGAAACCATGG |
|  | *CSUGT74B5-PBTEX*-R | AACGTCGTATGGGTAAGGCCTATAATGACCATTTCCATTGG |
| Construction of  subcellular localization vectors | *CsUGT74B5-eGFP*-F | CGAACGATAGCCATGGTACCAATGGAGCAAAGAAACCATGG |
|  | *CsUGT74B5-eGFP*-R | CATGCCTGCGGCCGCGCCGGATCCATAATGACCATTTCCATTGG |
| Construction of heterologous expression vectors | *CsUGT74B5+attb-*F | GGGGACAAGTTTGTACAAAAAAGCAGGCTATGGAGCAAAGAAACCAT |
|  | *CsUGT74B5+attb-*R | GGGGACCACTTTGTACAAGAAAGCTGGGTTCAATAATGACCATTTCC |

**Table S2.** Sequences of primers used for quantitative RT-PCR.

| Purpose | Gene name | Primer name | Primer sequence (5'-3') |
| --- | --- | --- | --- |
| Quantitative  RT-PCR | *CsUGT74B5* | *CsUGT74B5*-qRT-F | AAGCCAATCCGTCGTCTATGT |
|  |  | *CsUGT74B5*-qRT-R | CTCACCACCCAAATGAAGTCC |
|  | *CsUGT78A15* | *CsUGT78A15*-qRT-F | TTGGTGTGAGAGTTGAGGG |
|  |  | *CsUGT78A15*-qRT-R | GGTCCAACAGCCTTGAGAG |
|  | *CsUGT75L43* | *CsUGT75L43*-qRT-F | GCACCCGTCTTTGGGATGCTTC |
|  |  | *CsUGT75L43*-qRT-R | AGCCTCCAATGTTGGCATGT |
|  | *CsDMR6-1* | *CsDMR6-1*-qRT-F | CTCAAGGACGGCAAGTGGA |
|  |  | *CsDMR6-1*-qRT-R | ATGGCTCGGTGCCATACAC |
|  | *CsDMR6-2* | *CsDMR6-2*-qRT-F | ATGCCCAGAACCAGAGTTGAC |
|  |  | *CsDMR6-2*-qRT-R | ATGGCTCGGTGCCATACAC |
|  | *CsSAMT* | *CsSAMT*-qRT-F | AGAGTGTTGTTACATTTGGGAGC |
|  |  | *CsSAMT*-qRT-R | CTACACCGAAATGACTAACAAGC |
|  | *CsUGT87E7* | *CsUGT87E7*-qRT-F | TTTCCCATTTATTCTTTCGG |
|  |  | *CsUGT87E7*-qRT-R | TTTAACCTGGACGCTTTGT |
|  | *CsPR1* | *CsPR1*-qRT-F | GACAGTGTCGGATGCAGTGA |
|  |  | *CsPR1*-qRT-R | TTCTGGCACTGAACCCTAGC |
|  | *CsPR2* | *CsPR2*-qRT-F | CTCTGTTCACAGCTCCCTCG |
|  |  | *CsPR2*-qRT-R | GTTCCACCAACTGTAGGCCA |
|  | *CsGAPDH* | *CsGAPDH*-qRT-F | TTGGCATCGTTGAGGGTCT |
|  |  | *CsGAPDH*-qRT-R | CAGTGGGAACACGGAAAGC |
|  | *AtGAPDH* | *AtGAPDH*-qRT-F | CAACCGGTATTGTGCTGGATTC |
|  |  | *AtGAPDH*-qRT-R | ATCCACATCTGCTGGAATGTGC |
|  | *NtGAPDH* | *NtGAPDH*-qRT-F | TAGAAACCCCAAGTACCCTCG |
|  |  | *NtGAPDH*-qRT-R | TGCTTTCTTCGTCCCATCAG |

**Table S3.** The binding sites of ligands in CsUGT74B5 protein.

| Protein-Ligand | Minimum binding energy/(kcal/mol) | Binding sites of ligands |
| --- | --- | --- |
| CsUGT74B5-SA | -5.27 | Phe-276, Trp-303, Val-304 and Arg-306 |
| CsUGT74B5-2,6-DHBA | -5.12 | Phe-276, Trp-303, Val-304 and Arg-306, Gly-277 |
| CsUGT74B5-2,5-DHBA | -4.03 | Gln-269, Leu-361, Gly-362, Lys-428 |
| CsUGT74B5-3,4-DHBA | -4.73 | Ser-278, Met-279, Trp-352, Gln-374, Asp-373 |
